# Hepatic Steatosis Complicating Pediatric Idiopathic Nephrotic Syndrome During Systemic Corticosteroid Therapy: A Case Report

**DOI:** 10.7759/cureus.114342

**Published:** 2026-08-11

**Authors:** Masaaki Doi, Naohiro Akisada, Yasuko Furuichi

**Affiliations:** 1 Pediatrics, Higashiosaka City Medical Center, Higashiosaka, JPN; 2 Pediatrics, Minaminara General Medical Center, Oyodo-cho, JPN; 3 Pediatrics, Nara Medical University, Kashihara, JPN

**Keywords:** case report, corticosteroids, hepatic steatosis, hyperlipidemia, nephrotic syndrome, ultrasonography

## Abstract

Hepatic steatosis is a rare complication of pediatric idiopathic nephrotic syndrome, and its clinical course has infrequently been described. A 22-month-old boy presented with generalized edema, oliguria, and massive proteinuria and was diagnosed with idiopathic nephrotic syndrome complicated by urinary tract infection. Following infection control, prednisolone was initiated according to the International Study of Kidney Disease in Children regimen, and complete remission was achieved by day 17. Serum aminotransferase levels increased on day 19, followed by progressive hepatomegaly. Abdominal ultrasonography revealed mild hepatic steatosis (score 1) on day 28, which progressed to severe steatosis (score 3) by day 45 despite improving serum lipid and aminotransferase levels. Noncontrast computed tomography on day 37 revealed hepatomegaly with decreased hepatic attenuation. A workup for secondary causes of steatosis was unremarkable. Following prednisolone tapering and discontinuation, hepatomegaly resolved by day 64, and hepatic steatosis gradually improved, with normalization confirmed on ultrasonography on day 246. The nephrotic syndrome has not recurred for more than five years after corticosteroid withdrawal. Hepatic steatosis may develop during corticosteroid treatment for pediatric idiopathic nephrotic syndrome, potentially owing to nephrotic hyperlipidemia and/or corticosteroid exposure. Ultrasonographic recovery may lag behind clinical remission and may take several months.

## Introduction

Idiopathic nephrotic syndrome (INS), the most common form of pediatric nephrotic syndrome, is characterized by massive proteinuria with hypoalbuminemia and hyperlipidemia, and systemic corticosteroids remain the first-line therapy for inducing remission [1].

Although metabolic dysfunction-associated steatotic liver disease (MASLD) related to cardiometabolic risk factors (e.g., obesity, insulin resistance, and hypertension) is increasingly recognized in children [2-4], secondary steatosis may also develop in conjunction with endocrine disorders, inborn errors of metabolism, viral hepatitis, and exposure to steatogenic medications [5].

Hepatic steatosis has been described in patients with nephrotic syndrome, albeit rarely in pediatric practice [6,7]. Mechanistically, significant nephrotic hyperlipidemia and systemic corticosteroid therapy can potentially contribute to hepatic lipid accumulation [5,8]. However, the clinical course of hepatic steatosis developing in pediatric patients with INS has rarely been documented [6,7].

We report the case of a 22-month-old boy with pediatric INS complicated by hepatic steatosis. The steatosis resolved following nephrotic syndrome remission and systemic corticosteroid withdrawal.

## Case presentation

The patient was a 22-month-old boy with no significant past medical history and was not taking any medications. Newborn mass screening for inborn errors of metabolism was unremarkable. No family history of dyslipidemia, coronary artery disease, or stroke was documented. He developed periorbital edema and fever on day 0, followed by generalized edema on day 3. On day 7, he was referred to Higashiosaka City Medical Center (Osaka, Japan) owing to marked edema, oliguria, and 4+ proteinuria on a urine dipstick test performed at a local clinic.

On admission (day 7), his body weight was 11.5 kg (usual, 9.6 kg; +18.9%; standard deviation (SD), −1.0), and his height was 82 cm (SD, −0.8), yielding a body surface area of 0.46 m². His temperature, SpO₂, heart rate, and blood pressure were 35.2 °C, 98% on room air, 105 beats/min, and 100/50 mmHg, respectively. He appeared lethargic, with cold extremities. No rash or purpura was noted.

Urinalysis revealed massive proteinuria (urine protein-to-creatinine ratio, 23.29 g/gCr) and pyuria (5-9 white blood cells/high-power field) without hematuria. Blood tests demonstrated leukocytosis with neutrophil predominance (white blood cell count, 27,530/μL; neutrophils, 72%), hypoproteinemia (total protein, 3.0 g/dL) with marked hypoalbuminemia (0.8 g/dL), hyponatremia (118 mEq/L), and an elevated C-reactive protein level (10.6 mg/dL). Serum triglyceride, total cholesterol, and blood urea nitrogen levels were elevated. Immunoglobulin (Ig) testing revealed low IgG and IgA levels. Liver enzyme levels were within the reference ranges, with an aspartate aminotransferase (AST) level of 38 U/L and an alanine aminotransferase (ALT) level of 11 U/L. Catheterized urine culture yielded *Enterococcus faecalis* at 4 × 10⁴ colony-forming units/mL, whereas blood cultures were negative. Laboratory and microbiological findings on admission are summarized in Table 1.

**Table 1:** Laboratory and microbiological findings on admission Reference values for serum creatinine, marked with a superscript dagger (†), are based on reference [9]. Normal values for all other parameters represent institutional reference ranges. AST, aspartate aminotransferase; ALT, alanine aminotransferase; LDH, lactate dehydrogenase; IgG, immunoglobulin G; IgA, immunoglobulin A; IgM, immunoglobulin M; C3, complement component 3; C4, complement component 4; CH50, total hemolytic complement activity; BUN, blood urea nitrogen; CRP, C-reactive protein; HPF, high-power field; CFU, colony-forming units.

Examinations	Patient	Normal values
Red blood cells (/μL)	4.83 x 10^6^	4.38–5.77 x 10^6^
White blood cells (/μL)	27,530	3,500–9,700
Neutrophils (% leukocytes)	72	42–74
Platelets (/μL)	514 x 10^3^	140–379 x 10^3^
Total protein (g/dL)	3.0	6.5–8.2
Albumin (g/dL)	0.8	3.7–5.5
AST (IU/L)	38	10–40
ALT (IU/L)	11	5–45
LDH (IU/L)	497	120–245
IgG (mg/dL)	65	820–1740
IgA (mg/dL)	39	90–400
IgM (mg/dL)	92	31–200
C3 (mg/dL)	112	80–140
C4 (mg/dL)	15	11.0–34.0
CH50 (CH50/mL)	25	25.0–48.0
Antinuclear antibody titer	< 1:20	< 1:40
Total cholesterol (mg/dL)	293	150–219
Triglycerides (mg/dL)	318	50–149
BUN (mg/dL)	36.9	8–20
Creatinine (mg/dL)	0.33	0.16–0.32^†^
Sodium (mEq/L)	118	135–145
Potassium (mEq/L)	5.2	2.5–4.5
Chloride (mEq/L)	87	98–108
Calcium (mg/dL)	6.5	8.6–10.2
CRP (mg/dL)	10.63	< 0.30
Urine		
Appearance	Turbid (+)	Clear (−)
Urine color	Pale yellow	
Urine dipstick		
Specific gravity	1.025	1.008–1.034
pH	5.5	4.8–7.5
Urobilinogen	+/−	Negative (−) to trace
Protein (dipstick)	4+	Negative (−) to trace
Occult blood (dipstick)	−	Negative (−)
Glucose (dipstick)	−	Negative (−) to trace
Ketones (dipstick)	−	Negative (−)
Nitrite (dipstick)	+	Negative (−)
Urinalysis		
Total protein (mg/dL)	965	< 10
Urine protein-to-creatinine ratio (g/gCr)	23.29	< 0.15
Red blood cells (/HPF)	1–4	< 5
White blood cells (/HPF)	5–9	< 5
β2-microglobulin (μg/L)	214	< 230
Culture		
Urine culture	Enterococcus faecalis 4 × 10^4^ CFU/mL	
Blood culture	No growth	

Abdominal ultrasonography revealed mild bilateral renal enlargement with slight dilatation of the renal pelves, without apparent liver abnormalities; hepatorenal echo contrast was negative, and neither ascites nor pleural effusion was detected. Echocardiography demonstrated preserved cardiac function without pericardial effusion; the inferior vena cava appeared collapsed. Chest radiography revealed no significant findings; the cardiothoracic ratio was within the normal range at 0.52 [10]. Based on massive proteinuria with severe hypoalbuminemia and generalized edema, he was diagnosed with pediatric INS complicated by urinary tract infection [11].

As none of the features suggestive of a nonminimal change histology were present, such as age younger than one year, persistent microscopic hematuria or gross hematuria, hypertension and/or impaired renal function, hypocomplementemia, or extrarenal manifestations (e.g., rash or purpura), we judged the presentation to be most consistent with presumed minimal change disease and steroid-sensitive nephrotic syndrome and therefore did not perform a kidney biopsy [11].

Following admission (day 7), intravenous isotonic saline and albumin (1 g/kg) were administered to correct the decreased effective circulating plasma volume. Cefotaxime sodium (100 mg/kg/day, days 7-11) and intravenous Ig (215 mg/kg, days 7-9) were administered for managing the urinary tract infection. On day 8, his fever resolved; on day 9, his inflammatory markers improved (peripheral white blood cell count, 8,150/μL; neutrophils, 31%; and C-reactive protein, 0.29 mg/dL). Therefore, prednisolone therapy was initiated according to the International Study of Kidney Disease in Children regimen [11]. On day 9, intravenous prednisolone at 60 mg/m²/day was initiated. On day 11, urinary protein excretion began to decrease. On day 14, prednisolone was switched from intravenous to oral administration; on day 17, complete remission was achieved. No other medications were administered during this treatment period.

**Figure 1:**
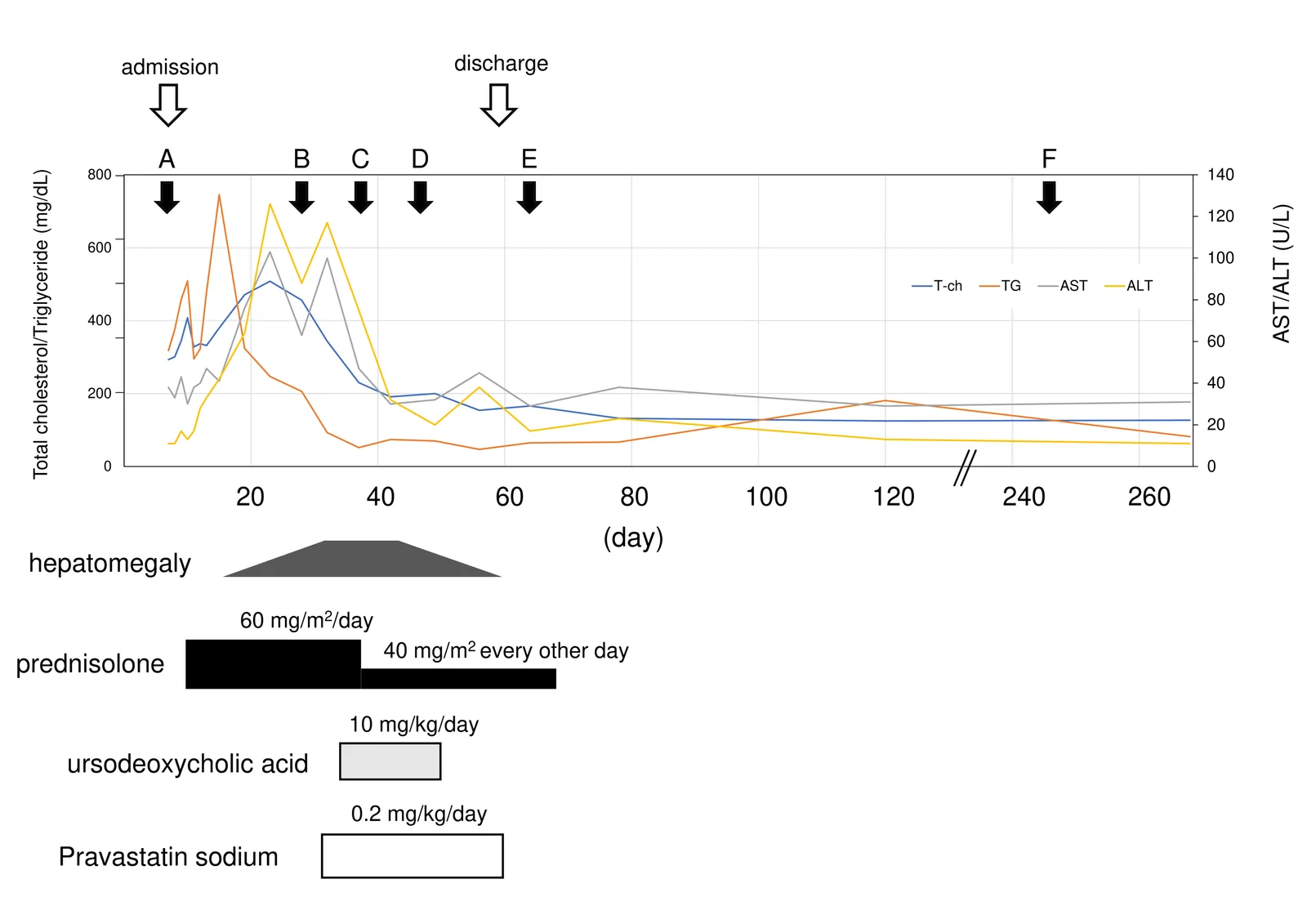
Clinical course of serum lipid levels, aminotransferase levels, hepatomegaly, and treatment Time course of serum lipids and liver enzymes: serum total cholesterol and triglycerides are plotted on the primary y-axis, and AST and ALT on the secondary y-axis. The degree of hepatomegaly and the treatment course (prednisolone, ursodeoxycholic acid, and pravastatin) are shown below the graph. White arrows indicate admission and discharge. Black arrows labeled A–F indicate the timing of abdominal ultrasonography corresponding to the images shown in Figure 2. The x-axis is truncated after day 120 (axis break). AST, aspartate aminotransferase; ALT, alanine aminotransferase; TG, triglycerides; T-chol, total cholesterol.

**Figure 2:**
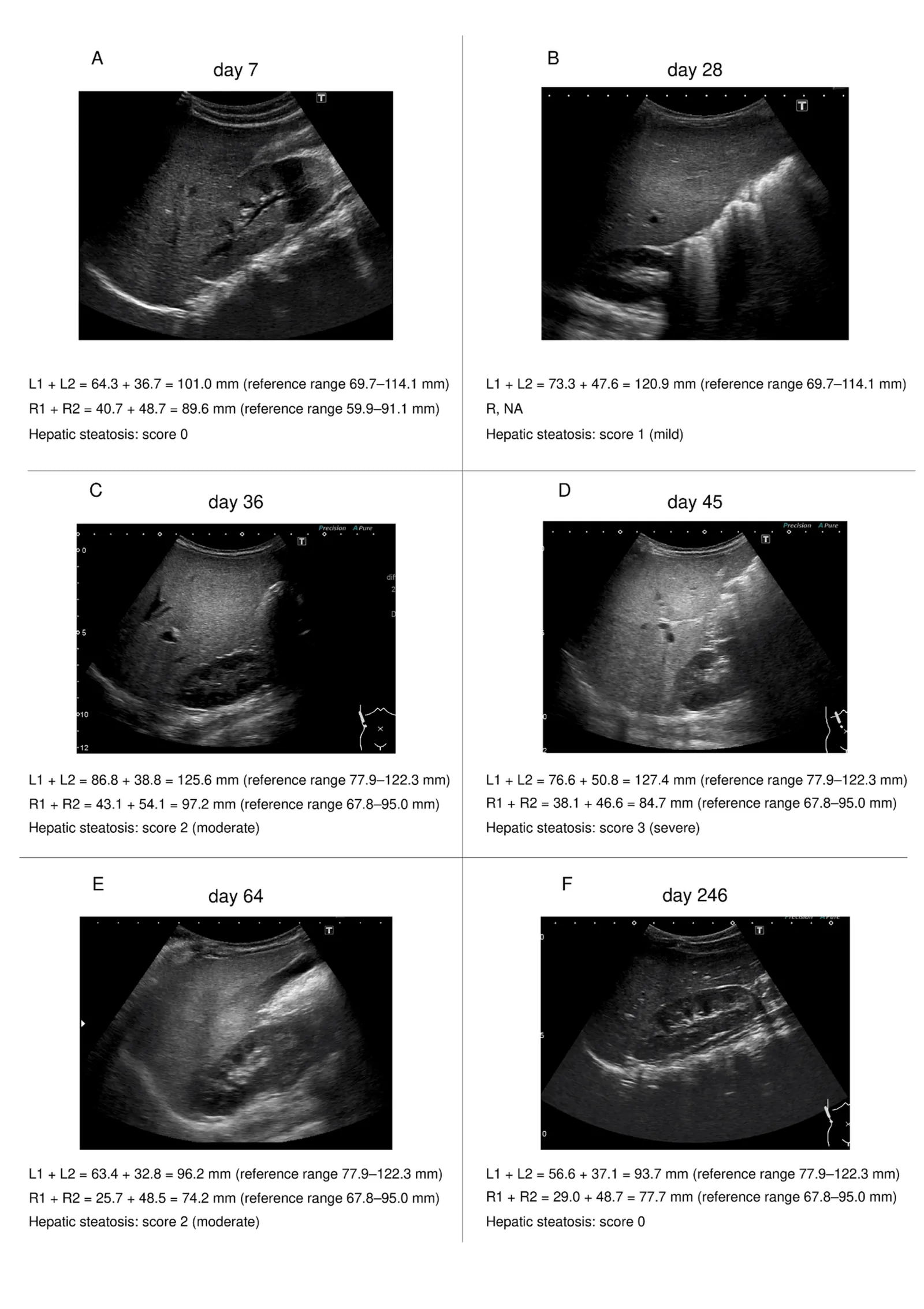
Serial abdominal ultrasonography findings of hepatic steatosis Representative B-mode liver ultrasonography images at serial time points: panels A-F correspond to the abdominal ultrasonography time points labeled A–F in Figure 1. For each image, the steatosis grade and corresponding liver size are indicated below the panel. Steatosis was graded based on hepatic echogenicity using the semiquantitative B-mode ultrasonographic grading system for liver steatosis described by Ferraioli and Soares Monteiro [13]. Grade 0 indicates no increase in hepatic echogenicity; grade 1, mild diffuse increase in echogenicity with preserved visualization of the diaphragm and portal vein walls; grade 2, moderate increase in echogenicity with partial loss of clarity of the diaphragm and portal vein walls; and grade 3, marked increase in echogenicity with pronounced posterior attenuation and poor visualization of the portal vein walls, diaphragm, and posterior right lobe.

**Figure 3:**
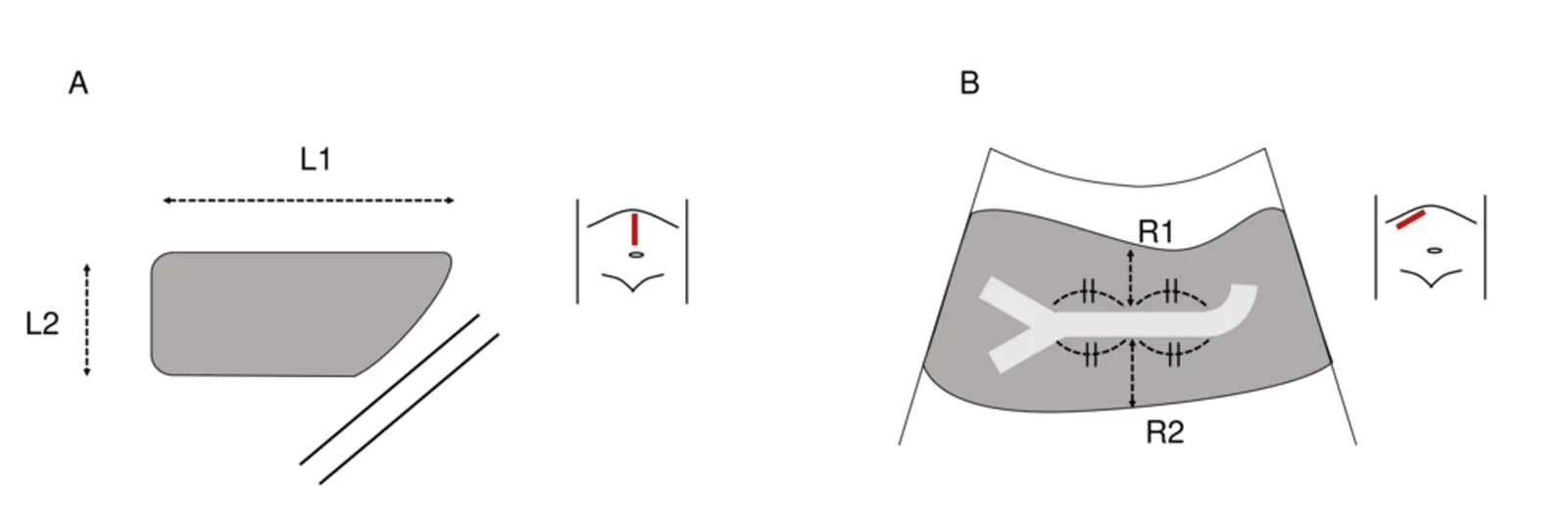
Schematic of ultrasonographic liver-size measurements The schematic was created by the authors using Microsoft PowerPoint (Microsoft Corporation, Redmond, WA, US) and was not generated using artificial intelligence. The measurement method is based on Yokota et al. [12]. (A) Left lobe measurement was performed on a longitudinal scan above the aorta: L1, maximal long-axis length; L2, maximal depth. (B) Right lobe measurement was performed on a subcostal scan through the hepatic hilum: R1, shortest distance from the anterior liver surface to the midpoint of the horizontal portal vein; R2, distance from that midpoint to the deepest diaphragmatic surface. Liver size was calculated as L = L1 + L2 and R = R1 + R2 and compared with age-matched Japanese reference ranges (±2 SD) from the same reference.

However, on day 19, serum aminotransferase levels increased (AST/ALT, 76/64 U/L) and remained elevated thereafter. Hepatomegaly gradually progressed; on day 25, the liver became palpable three fingerbreadths below the costal margin. On day 28, abdominal ultrasonography revealed hepatomegaly based on the age-matched ultrasonographic liver-size reference ranges described by Yokota et al. [12]. The liver showed a mild, diffuse increase in hepatic echogenicity, whereas visualization of the portal vein walls and the diaphragm was preserved, consistent with mild hepatic steatosis, corresponding to score 1 on the semiquantitative B-mode ultrasonographic grading system for liver steatosis described by Ferraioli and Soares Monteiro [13]. On day 36, follow-up ultrasonography demonstrated progression to moderate steatosis (score 2). On day 37, noncontrast computed tomography revealed hepatomegaly with decreased hepatic attenuation (27 Hounsfield units), supporting the diagnosis of hepatic steatosis in the setting of INS. Serial changes in serum lipid levels, aminotransferase levels, hepatomegaly, and treatment are shown in Figure 1. Serial abdominal ultrasonography findings are shown in Figure 2. The ultrasonographic liver-size measurement method is illustrated in Figure 3.

Laboratory workup for potential secondary causes of hepatic steatosis was performed on days 28-37. The findings are summarized in Table 2.

**Table 2:** Workup for potential secondary causes of hepatic steatosis AFP, alpha-fetoprotein; CMV, cytomegalovirus; EBV, Epstein–Barr virus; EBNA, Epstein–Barr nuclear antigen; VCA, viral capsid antigen; LKM-1, liver–kidney microsomal type 1 antibody. The EBV serologic profile (VCA IgG positive with VCA IgM negative and EBNA positive) was consistent with a past EBV infection. Values preceded by “<” or reported as “undetectable” indicate results below the assay detection limit. Plasma amino acid analysis (39 analytes) showed no abnormal findings. Normal values represent institutional reference ranges.

Examinations	Patient	Normal values
EBV-VCA IgG	10	<10
EBV-VCA IgM	<10	<10
EBNA	10	<10
CMV-IgG (AU/mL)	<2	<6
CMV-IgM	<0.80	<0.85
Hepatitis B surface antigen	Negative	Negative
Hepatitis C virus antibody	Negative	Negative
Lactate (mg/dL)	25.6	3–17
Pyruvate (mg/dL)	2.2	0.30–0.94
AFP (ng/mL)	4.4	<10
Total bile acids (µmol/L)	1.1	<10
Anti-LKM-1 antibody	Undetectable	<17
Plasma amino acid analysis (39 analytes)	No abnormal findings	

Serologic testing for Epstein-Barr virus (EBV) revealed previous infection (viral capsid antigen IgG, 10; viral capsid antigen IgM, <10; and Epstein-Barr nuclear antigen, 10), whereas serologic testing for cytomegalovirus (CMV) showed no evidence of past or current infection. Tests for hepatitis B surface antigen and hepatitis C virus antibody were negative. Serum lactate and pyruvate levels were elevated at 25.6 (reference range, 3-17) and 2.2 (0.30-0.94) mg/dL, respectively. Alpha-fetoprotein and total bile acids were within normal limits, and the anti-liver-kidney microsomal type 1 antibody test was negative. Plasma amino acid analysis (39 analytes) revealed no abnormalities. 

Oral pravastatin sodium (0.2 mg/kg/day) and ursodeoxycholic acid (10 mg/kg/day) were initiated on days 29 and 32, respectively; pravastatin and ursodeoxycholic acid were discontinued on days 59 and 49, respectively. On day 38, prednisolone was tapered to 40 mg/m² every other day. Serum total cholesterol and triglyceride levels remained markedly elevated during the early phase of treatment but subsequently decreased and normalized on days 42 and 32, respectively. Serum aminotransferase levels began decreasing on day 32 and normalized on day 42. The patient was discharged on day 58, and prednisolone was discontinued on day 66. The severity of hepatic steatosis was monitored using serial abdominal ultrasonography. Despite improvement in serum lipid levels, ultrasonographic findings initially worsened, reaching severe steatosis (score 3) on day 45, and then gradually improved. Furthermore, hepatomegaly progressively resolved, with liver size normalizing by day 64. Hepatic steatosis persisted at least through day 64, and abdominal ultrasonography on day 246 confirmed its resolution; no ultrasonography was performed between days 64 and 246. To date, his nephrotic syndrome has not recurred for more than five years after prednisolone discontinuation.

## Discussion

This case underscores two significant clinical points: hepatic steatosis may develop in pediatric INS during systemic corticosteroid therapy, potentially owing to nephrotic hyperlipidemia and/or corticosteroid exposure; and hepatic steatosis may spontaneously resolve following nephrotic syndrome remission and corticosteroid discontinuation, although ultrasonographic recovery may take several months.

Pediatric hepatic steatosis has a broad differential diagnosis. It can be broadly categorized as either MASLD associated with cardiometabolic risk factors or as secondary steatosis related to metabolic disorders, viral hepatitis, or steatogenic medications, including systemic corticosteroids [2,4,5]. Hepatic steatosis is a recognized but uncommon complication of nephrotic syndrome in children [6], and experimental and clinical findings suggest that severe nephrotic hyperlipidemia and systemic corticosteroid therapy can promote hepatic lipid deposition [8,14,15].

Nephrotic syndrome is characterized by severe hyperlipidemia due to dysregulated hepatic lipid metabolism [11,16]. In the nephrotic state, hepatic production of lipoproteins (e.g., very-low-density and low-density lipoproteins) and lipid synthesis are increased, whereas lipoprotein clearance and fatty acid oxidation may be relatively reduced [16,17], causing significantly elevated circulating cholesterol and triglyceride levels that can contribute to hepatic lipid accumulation. Clinically, patients with nephrotic syndrome exhibit a higher prevalence of hepatic steatosis even after adjustment for major metabolic risk factors, including obesity and diabetes [15]. This finding suggests that nephrotic syndrome itself, including nephrotic hyperlipidemia, may contribute to the development of hepatic steatosis independently of conventional metabolic risk factors.

Moreover, systemic glucocorticoids can promote hepatic triglyceride accumulation through several complementary mechanisms. They enhance gluconeogenesis and peripheral lipolysis in the fasted state, increasing free fatty acid delivery to the liver. In the fed state, glucocorticoids potentially act synergistically with insulin to stimulate hepatic de novo lipogenesis [8]. Experimental studies have further suggested that glucocorticoids can increase hepatic triglyceride synthesis and fatty acid uptake while reducing fatty acid oxidation, thereby shifting the balance toward hepatic lipid storage [8].

In our patient, other potential causes of hepatic steatosis were considered less likely. No family history of inherited disorders or dyslipidemia was reported, and there were no clinical features indicating chronic liver disease or hepatomegaly before nephrotic syndrome onset. Hepatitis B surface antigen and hepatitis C virus antibody tests were negative, and serology indicated a previous EBV infection without evidence of active CMV infection. The alpha-fetoprotein level was within normal limits, and the anti-liver-kidney microsomal type 1 antibody test was negative. Metabolic screening, including serum lactate, pyruvate, and plasma amino acid analysis, did not provide evidence supporting mitochondrial disease or other inborn errors of metabolism. Although lactate and pyruvate levels were slightly elevated, no clinical features (e.g., neurologic manifestations or episodic decompensation) were noted suggestive of a primary disorder of pyruvate metabolism, rendering this etiology less likely. Genetic testing for inherited metabolic or lipid disorders was not performed because the patient had no suggestive family history or clinical features and the hepatic steatosis completely resolved during follow-up. Nevertheless, the absence of genetic testing is a limitation of this report.

Apart from systemic corticosteroids, no other medications known to be associated with hepatic steatosis were administered. Collectively, and considering the temporal association with nephrotic hyperlipidemia and corticosteroid therapy, secondary hepatic steatosis associated with the nephrotic state and corticosteroid exposure was the most plausible explanation in this case.

A comprehensive previous report describing pediatric nephrotic syndrome-associated hepatic steatosis [7] noted that fatty liver developed approximately two weeks following the onset of nephrotic syndrome and initiation of corticosteroid therapy. Following remission and corticosteroid withdrawal, hepatic enlargement and echogenicity progressively improved, with complete normalization occurring at approximately two months following discontinuation of therapy. Another observational study of children with nephrotic syndrome managed with prednisolone reported that hepatomegaly was detected several weeks after treatment initiation and began to regress only after steroid tapering and clinical remission, with normalization reported within 2-6 months. Although based on palpation rather than imaging, this clinical course also suggests a temporal delay between disease improvement and the resolution of hepatic changes [6]. Similarly, in corticosteroid-induced hepatic steatosis, although simple steatosis frequently rapidly resolves, steatohepatitis may take longer to improve, suggesting that radiologic abnormalities can persist following drug withdrawal [18].

In the present case, hepatic steatosis followed a comparable time course, that is, emerging following nephrotic syndrome onset and corticosteroid initiation and persisting even after clinical remission and tapering of therapy. The subsequent gradual resolution of steatosis further supports the possibility that nephrotic hyperlipidemia and/or corticosteroid exposure contributed to its development. Notably, the imaging-based appearance and resolution of hepatic steatosis lagged behind the clinical course of the nephrotic syndrome. This temporal dissociation potentially reflects the underlying pathophysiological sequence: sustained hyperlipidemia may necessitate an extended time to induce lipid accumulation in hepatocytes and likewise for hepatic lipid content and liver size to normalize after systemic lipid metabolism returns to baseline.

## Conclusions

This case illustrates that hepatic steatosis can occur during systemic corticosteroid therapy for pediatric idiopathic nephrotic syndrome, potentially in association with nephrotic hyperlipidemia and/or corticosteroid exposure. In the present patient, hepatic steatosis progressed despite improvement in serum lipid and aminotransferase levels, suggesting that imaging findings may lag behind biochemical and clinical recovery.

Clinicians should consider secondary hepatic steatosis when hepatomegaly or aminotransferase elevation develops during treatment for pediatric nephrotic syndrome, after excluding other relevant causes. Serial ultrasonography may be useful for monitoring the course of hepatic steatosis, and families should be informed that radiologic normalization may require several months even after remission of nephrotic syndrome and corticosteroid withdrawal.

## References

[1] Vivarelli M, Gibson K, Sinha A, Boyer O (2023). Childhood nephrotic syndrome. Lancet.

[2] Goyal NP, Xanthakos S, Schwimmer JB (2025). Metabolic dysfunction-associated steatotic liver disease in children. Gut.

[3] Rinella ME, Lazarus JV, Ratziu V (2023). A multisociety Delphi consensus statement on new fatty liver disease nomenclature. Hepatology.

[4] Vos MB, Abrams SH, Barlow SE (2017). NASPGHAN clinical practice guideline for the diagnosis and treatment of nonalcoholic fatty liver disease in children: recommendations from the expert committee on NAFLD (ECON) and the North American Society of Pediatric Gastroenterology, Hepatology and Nutrition (NASPGHAN). J Pediatr Gastroenterol Nutr.

[5] Liebe R, Esposito I, Bock HH (2021). Diagnosis and management of secondary causes of steatohepatitis. J Hepatol.

[6] Bhakoo ON, Walia BN (1963). Fatty liver in prednisolone treated nephrotic children. J Trop Pediatr Afr Child Health.

[7] Huttunen NP, Anttinen J, Kaira P, Koskimies O (1997). An 8-year-old girl with nephrotic syndrome, delayed response to prednisolone, and fatty liver. Pediatr Nephrol.

[8] Woods CP, Hazlehurst JM, Tomlinson JW (2015). Glucocorticoids and non-alcoholic fatty liver disease. J Steroid Biochem Mol Biol.

[9] Uemura O, Honda M, Matsuyama T (2011). Age, gender, and body length effects on reference serum creatinine levels determined by an enzymatic method in Japanese children: a multicenter study. Clin Exp Nephrol.

[10] Keats TE, Lusted LB, Lusted EB (1985). Atlas of Roentgenographic Measurement, 5th ed.

[11] (2026). Japanese Society for Pediatric Nephrology. Clinical practice guideline for pediatric idiopathic nephrotic syndrome 2020. https://minds.jcqhc.or.jp/common/summary/pdf/c00605.pdf.

[12] Yokota K, Wang Y, Ono T (2001). Use of ultrasonography to measure liver size in children. J Med Ultrasonics.

[13] Ferraioli G, Soares Monteiro LB (2019). Ultrasound-based techniques for the diagnosis of liver steatosis. World J Gastroenterol.

[14] Toblli JE, Ferder L, Stella I, Angerosa M, Inserra F (2002). Enalapril prevents fatty liver in nephrotic rats. J Nephrol.

[15] Onwuzo SS, Hitawala AA, Boustany A (2023). Prevalence of non-alcoholic fatty liver disease in patients with nephrotic syndrome: a population-based study. World J Hepatol.

[16] Agrawal S, Zaritsky JJ, Fornoni A, Smoyer WE (2018). Dyslipidaemia in nephrotic syndrome: mechanisms and treatment. Nat Rev Nephrol.

[17] Zhou Y, Zhang X, Chen L (2008). Expression profiling of hepatic genes associated with lipid metabolism in nephrotic rats. Am J Physiol Renal Physiol.

[18] National Institute of Diabetes and Digestive and Kidney Diseases (2026). Corticosteroids. LiverTox: Clinical and Research Information on Drug-Induced Liver Injury [Internet].

